# The Role of Cytochrome P450 Enzymes in COVID-19 Pathogenesis and Therapy

**DOI:** 10.3389/fphar.2022.791922

**Published:** 2022-02-02

**Authors:** Guyi Wang, Bing Xiao, Jiayi Deng, Linmei Gong, Yi Li, Jinxiu Li, Yanjun Zhong

**Affiliations:** ^1^ Department of Critical Care Medicine, The Second Xiangya Hospital, Central South University, Changsha, China; ^2^ Department of Emergency, The Second Xiangya Hospital, Central South University, Changsha, China; ^3^ Department of Cardiology, The Second Xiangya Hospital, Central South University, Changsha, China

**Keywords:** COVID-19, SARS-CoV-2, CYP = cytochrome P450, inflammation, drug metabolism

## Abstract

Coronavirus disease 2019 (COVID-19) has become a new public health crisis threatening the world. Dysregulated immune responses are the most striking pathophysiological features of patients with severe COVID-19, which can result in multiple-organ failure and death. The cytochrome P450 (CYP) system is the most important drug metabolizing enzyme family, which plays a significant role in the metabolism of endogenous or exogenous substances. Endogenous CYPs participate in the biosynthesis or catabolism of endogenous substances, including steroids, vitamins, eicosanoids, and fatty acids, whilst xenobiotic CYPs are associated with the metabolism of environmental toxins, drugs, and carcinogens. CYP expression and activity are greatly affected by immune response. However, changes in CYP expression and/or function in COVID-19 and their impact on COVID-19 pathophysiology and the metabolism of therapeutic agents in COVID-19, remain unclear. In this analysis, we review current evidence predominantly in the following areas: firstly, the possible changes in CYP expression and/or function in COVID-19; secondly, the effects of CYPs on the metabolism of arachidonic acid, vitamins, and steroid hormones in COVID-19; and thirdly, the effects of CYPs on the metabolism of therapeutic COVID-19 drugs.

## 1 Introduction

Coronavirus disease 2019 (COVID-19), which is caused by severe acute respiratory syndrome coronavirus 2 (SARS-CoV-2), has become a global challenge. As of December 29, 2021, there have been over 281 million confirmed cases of COVID-19, including more than five million deaths, reported to the WHO (WHO, 2021). Numerous SARS-CoV-2 variants have been detected around the world. Many SARS-CoV-2 variants are more infectious than original wild strain, which have brought new challenges to the prevention and control of COVID-19 (Tian et al., 2021). Dysregulated immune response, particularly cytokine storm, is a prominent feature of COVID-19, which can result in multiple-organ failure and death. The cytochrome P450 (CYP) enzymes form a large family of heme-containing enzymes that catalyze the metabolism of a variety of chemical compounds, and play a significant role in the metabolism of endogenous or exogenous substances. CYP expression and activity are greatly affected by immune mediators, such as interleukin (IL)-6, tumor necrosis factor (TNF)-α, IL-1, and interferon (IFN)-γ. However, changes in CYP expression and/or function in COVID-19 and their impact on its pathophysiology, and on the metabolism of therapeutic agents in COVID-19 remain unclear. This review focuses on the involvement of CYPs in the pathophysiology and pharmacotherapeutics of COVID-19.

## 2 Pathophysiological Characteristics of COVID-19

Dysregulated immune response is the most striking pathophysiological feature in severe COVID-19 patients; characterized by cytokine storm and lymphopenia; resulting in acute respiratory distress syndrome (ARDS), multiple-organ failure, and even death. SARS-CoV-2 may activate both innate and adaptive immune responses in patients, including lymphopenia, cytokine storm, and abnormal activation of macrophages and their complementary system (Qin et al., 2020; Tan et al., 2020; Xu et al., 2020; Jamal et al., 2021). Severe COVID-19 patients commonly exhibit a hyperinflammatory state referred to as cytokine storm, marked by elevation of IL-2, IL-4, IL-6, TNF-α, and IFN-γ (Copaescu et al., 2020; Hu et al., 2021; Paniri and Akhavan_Niaki, 2020; Qin et al., 2020; Zhang et al., 2021a). Elevated IL-6 concentration was shown to be associated with detectable serum SARS-CoV-2 RNA in patients with COVID-19 (Chen et al., 2020). A number of studies highlighted that elevation of IL-6 levels was correlated with adverse outcomes in SARS-CoV-2 infection, defined as severe COVID-19 occurrence, the requirement for mechanical ventilation, and death (Copaescu et al., 2020; Gao et al., 2020; Ruan et al., 2020; Belaid et al., 2021; Potere et al., 2021). IL-6 and IL-1 blockade may be associated with clinical improvement in patients with COVID-19 (Cavalli et al., 2020; Pinzon et al., 2021).

## 3 Cytochrome P450 Enzymes

**FIGURE 1:**
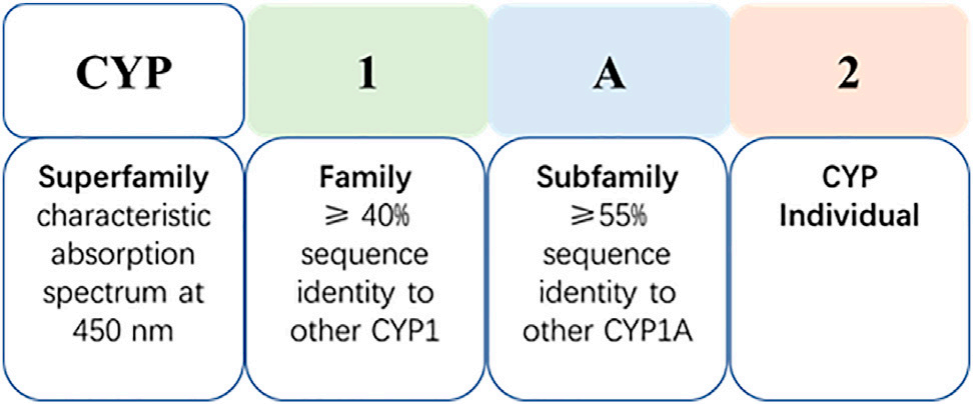
Cytochrome P450 nomenclature (take CYP1A2 as an example).

The CYP system is the most important drug metabolizing enzyme family existing amongst species, and plays a role in the metabolism of endogenous and exogenous substances (Stipp and Acco, 2021). CYP enzymes are located mainly within intestinal and hepatic tissues, but are also present in the skin, lung and kidneys etc. (He and Feng, 2015) There are 18 mammalian CYP families, located in the endoplasmic reticulum, or in mitochondrial membranes, which encode 57 genes in the human genome (Nebert et al., 2013; Korobkova, 2015; Stipp and Acco, 2021). CYP nomenclature reflects the characteristic absorption spectrum of the reduced enzyme at 450 nm, and the enzyme designation consists of a number-letter-number sequence on the basis of amino acid sequence homology (Figure 1).

CYPs are classified into two categories: endogenous (CYP family 7–51) and xenobiotic (CYP family 1–4) (Fan et al., 2016; Stipp and Acco, 2021). Endogenous CYPs participate in the biosynthesis, or catabolism of endogenous substance, whilst xenobiotic CYPs are associated with the metabolism of environmental toxins, drugs, and carcinogens. CYP1, CYP2, and CYP3 account for ∼75% of enzymes involved in the metabolism of all clinical use drugs and other xenobiotics (Guengerich, 2008; Stipp and Acco, 2021), whilst CYP4 is involved in eicosanoid metabolism. Nevertheless, several drug-metabolizing CYPs are also involved in the metabolism of endogenous compounds, such as CYP3A4 and CYP3A5.

CYP3A4, CYP2C9, CYP2C8, CYP1A2, and CYP2E1 are highly expressed in the liver, whilst, CYP2A6, CYP2D6, CYP2B6, CYP2C19, and CYP3A5 are less abundant in the liver. CYP2J2, CYP1A1, and CYP1B1 are mainly expressed extrahepatically (Zanger and Schwab, 2013; Stipp and Acco, 2021).

## 4 Changes in Cytochrome P450 Expression and/or Function in COVID-19

CYP gene expression is regulated by the activation of several nuclear receptors, including constitutive androstane receptor (CAR), pregnane X receptor (PXR) and aryl hydrocarbon receptor (AhR) (Moriya et al., 2012; Stipp and Acco, 2021). CYP expression and activity are also thought to be affected by multiple factors such as hormone levels, and environment, as well as pathological conditions such as infection, inflammation, and cancer (Morgan, 2009; Zanger and Schwab, 2013; Morgan, 2017; Esteves et al., 2021). Previous studies have shown that viral infection, inflammatory mediators and hepatic injury may affect the expression and activity of some CYPs, which are prevalent in COVID-19. Therefore, we attempted to explore the changes of CYPs in expression and/or function in COVID-19 patients.

### 4.1 Virus Infection

Until now, no studies have focused on the effects of SARS-CoV-2 on the expression and activity of CYPs. However, previous studies have found that CYPs expression changed in several viral infections. CYP1A1 activity was suppressed by 75% in coxsackievirus B3 infected mice (Funseth et al., 2002). CYP3A4 activity was suppressed in primary hepatocytes infected with adenovirus, and adenovirus-induced modification of PXR may be responsible for changes in hepatic CYP3A4 activity (Wonganan et al., 2014). CYP2A5 and CYP3A expression increased in hepatitis B virus (HBV)-transgenic mice (Kirby et al., 1994), whilst CYP2D6 expression decreased in hepatitis C virus (HCV) infected mice (Kikuchi et al., 2010).

### 4.2 Cytokines

Expression of CYPs is markedly regulated during inflammatory processes. *In vitro*, CYPs were regulated (nearly all down-regulation) by multiple cytokine treatments, including IL-6, TNF-α, IFN-γ, TGF-β and IL-1β (Table 1).

**TABLE 1:** The effect of cytokines on CYPs expression.

Cytokine	CYPs	Effects on CYPs	mRNA or protein or activity	Condition	Studies
IL-6	CYP1A1	↓	mRNA and protein	Human HepG2 hepatoma cells	Fukuda and Sassa (1994)
	CYP1A2	↓	mRNA	Hepatoma cells (HepG2, HepG2f and Hep3B)	Fukuda et al. (1992)
				Turpentine-induced aseptic inflammation in IL-6-deficient mice	Siewert et al. (2000)
				Human HepaRG hepatoma cell line	Rubin et al. (2015)
		↓	Activity	Human HepaRG hepatoma cell line	Rubin et al. (2015)
		↑	Activity	Clinical study in patients with active rheumatoid arthritis	Zhuang et al. (2015)
	CYP2A5	↓	mRNA	Turpentine-induced aseptic inflammation in IL-6-deficient mice	Siewert et al. (2000)
	CYP2A12	↓	Activity	IL-6 knockout mice after LPS administration	Warren et al. (2001)
	CYP2B6	↓	mRNA	Human primary hepatocytes	Aitken and Morgan (2007)
				Human HepaRG hepatoma cell line	Rubin et al. (2015)
		↓	Activity	Human HepaRG hepatoma cell line	Rubin et al. (2015)
	CYP2C8	↓	mRNA	Human primary hepatocytes	Aitken and Morgan (2007)
	CYP2C9	↓	mRNA	Human primary hepatocytes	Aitken and Morgan (2007)
			Activity	Clinical study in patients with active rheumatoid arthritis	Zhuang et al. (2015)
	CYP2C19	↓	mRNA	Human primary hepatocytes	Aitken and Morgan (2007)
			Activity	Clinical study in patients with active rheumatoid arthritis	Zhuang et al. (2015)
	CYP2E1	↓	mRNA	Human primary hepatocytes	Abdel-Razzak et al. (1993)
	CYP3 subfamily	↓	Activity	Clinical study in patients with active rheumatoid arthritis	Zhuang et al. (2015)
	CYP3A3	↓	mRNA	Hepatoma cells (HepG2, HepG2f and Hep3B)	Fukuda et al. (1992)
	CYP3A4	↓	mRNA	Both HepG2 and Caco-2 cells	Enokiya et al. (2021)
				Human primary hepatocytes	Aitken and Morgan (2007)
			Activity	Clinical study in patients with rheumatoid arthritis	Lee et al. (2017)
				Clinical study in patients with rheumatoid arthritis	Schmitt et al. (2011)
				Human HepaRG hepatoma cell line	Rubin et al. (2015)
	CYP3A5	↓	mRNA	Both HepG2 and Caco-2 cells	Enokiya et al. (2021)
	CYP3A11	↓	mRNA	Turpentine-induced aseptic inflammation in IL-6-deficient mice	Siewert et al. (2000)
TNF-α	CYP1A1	↓	mRNA and protein	Rat liver epithelial WB-F344 cells	Umannová et al. (2008)
	CYP1A2	↓	mRNA	Human primary hepatocytes	Dallas et al. (2012)
	CYP1B1	↑	mRNA and protein	Rat liver epithelial WB-F344 cells	Umannová et al. (2008)
	CYP2A4/5	↑	mRNA	C. rodentium mice model of infectious colitis	Nyagode et al. (2014)
	CYP2C8	↓	mRNA	Cynomolgus hepatocytes	Uno et al. (2020)
	CYP2C76	↓	mRNA	Cynomolgus hepatocytes	Uno et al. (2020)
	CYP2D6	↓	mRNA	Human primary hepatocytes	Dallas et al. (2012)
	CYP2E1	↓	mRNA	Human primary hepatocytes	Abdel-Razzak et al. (1993)
	CYP3A11	↓	mRNA	Mouse hepatocytes	Kinloch et al. (2011)
				C. rodentium mice model of infectious colitis	Nyagode et al. (2014)
	CYP3A25	↓	mRNA	Mouse hepatocytes	Kinloch et al. (2011)
				C. rodentium mice model of infectious colitis	Nyagode et al. (2014)
	CYP3A4	↓	mRNA	Human primary hepatocytes	Dallas et al. (2012)
IL-1	CYP1A1	↓	mRNA	Isolated rat hepatocytes	Barker et al. (1992)
				cultured rabbit hepatocytes	Calleja et al. (1997)
	CYP1A2	↓	mRNA	Isolated rat hepatocytes	Barker et al. (1992)
				cultured rabbit hepatocytes	Calleja et al. (1997)
IL-1β	CYP1A1	↓	mRNA	Cynomolgus hepatocytes	Uno et al. (2020)
	CYP1A2	↓	mRNA	Human primary hepatocytes	Abdel-Razzak et al. (1993)
	CYP2B6	↓	mRNA	Human hepatocytes	Assenat et al. (2004)
	CYP2C8	↓	mRNA	Cynomolgus hepatocytes	Uno et al. (2020)
	CYP2C9	↓	mRNA	Human hepatocytes	Assenat et al. (2004)
	CYP2C11	↓	mRNA	IL-1β-induced fevered rat	Kihara et al. (1998)
	CYP2C19	↓	mRNA	Cynomolgus hepatocytes	Uno et al. (2020)
	CYP2C76	↓	mRNA	Cynomolgus hepatocytes	Uno et al. (2020)
	CYP3A4	↓	mRNA	Human hepatocytes	Assenat et al. (2004)
	CYP3A5	↑	mRNA	Cynomolgus hepatocytes	Uno et al. (2020)
	CYP3A subfamily	↓	mRNA	IL-1β-induced fevered rat	Kihara et al. (1998)
IFN-γ	CYP1A2	↓	Protein	Human primary hepatocytes	Donato et al. (1997)
	CYP2B9	↓	mRNA	LPS-induced septic mice model	Nyagode et al. (2010)
	CYP2D9	↓	mRNA	C. rodentium-induced colitis mice model	Nyagode et al. (2010)
	CYP2D22	↓	mRNA	C. rodentium-induced colitis mice model; LPS-induced septic mice model	Nyagode et al. (2010)
	CYP2E1	↓	mRNA	LPS-induced septic mice model	Nyagode et al. (2010)
	CYP3A1	↓	mRNA	Rat primary hepatocytes	Tapner et al. (1996)
	CYP3A2	↓	mRNA	Rat primary hepatocytes	Tapner et al. (1996)
	CYP3A4	↓	Protein	Human primary hepatocytes	Donato et al. (1997)
	CYP3A11	↓	mRNA	C. rodentium-induced colitis mice model	Nyagode et al. (2010)
	CYP3A25	↓	mRNA	C. rodentium-induced colitis mice model	Nyagode et al. (2010)
	CYP4F18	↓	mRNA	C. rodentium-induced colitis mice model	Nyagode et al. (2010)

Abbreviations: COVID-19, coronavirus disease 2019; CYP, cytochrome P450; IL, interleukin; TNF, tumor necrosis factor; IFN, interferon; LPS, lipopolysaccharide. ↑ (Increased), ↓ (Reduced).

#### 4.2.1 Interleukin-6

IL-6 was considered to be the principal regulator of the hepatic acute-phase response. Previous studies have focused on investigating the effect of IL-6 on CYPs levels. When hepatoma cells were treated with IL-6, the levels of CYP1A1, CYP1A2, CYP2B6, CYP3A3, and CYP3A4 mRNAs were markedly suppressed, as well as activities of CYP1A2, CYP2B6, and CYP3A4 (Fukuda et al., 1992; Fukuda and Sassa, 1994; Rubin et al., 2015). In both HepG2 and Caco-2 cells, IL-6 also induced a significant concentration- and time-dependent decrease in CYP3A4 and CYP3A5 expression (Enokiya et al., 2021). In a rat hepatocyte and Kupffer cell co-culture (HKCC) model treated with trovafloxacin or acetaminophen, lipopolysaccharide (LPS) activation showed decreased IL-6 production with concomitant increases in CYP3A activity (Rose et al., 2016).

Additionally, CYP2A12 activity increased in IL-6 knockout mice after LPS administration compared to wild type (WT) mice (Warren et al., 2001). However, Siewert et al. showed that IL-6 was the major determinant in the down-regulation of CYP1A2, CYP2A5, and CYP3A11 in mice models of aseptic inflammation, whereas in the case of LPS-mediated septic mice models, the effects of IL-6 on CYP downregulation can be compensated by other cytokines (Siewert et al., 2000).

In human hepatocytes, IL-6 also decreases both rifampicin- and phenobarbital-mediated induction of CYP2B6, CYP2C8, CYP2C9, and CYP3A4, by negatively regulating PXR and CAR gene expression (Pascussi et al., 2000). Several other studies demonstrated that IL-6 induces drops in CYP2B6, CYP2C8, CYP2C9, CYP2C19, CYP2E1, and CYP3A4 mRNA levels in human hepatocytes, but studies on the effects of IL-6 on CYP1As expression have shown inconsistent results (Abdel-Razzak et al., 1993; Muntané-Relat et al., 1995; Aitken and Morgan, 2007; Dickmann et al., 2012). Jover et al. have explored the molecular mechanism of IL-6 regulation of CYP expression and demonstrated that IL-6 down-regulates CYP3A4 through translational induction of C/EBPβ-LIP (Jover et al., 2002).

Moreover, several clinical studies have demonstrated that IL-6 inhibitors enhance drug metabolism *via* CYP3A4, 2C9, and 2C19, but reduced the drug metabolism *via* CYP1A2 (Schmitt et al., 2011; Lee et al., 2017; White et al., 2021). Blocking IL-6 receptors, *via* the monoclonal antibodies tocilizumab and sarilumab has reversed CYP3A4 activity suppression in rheumatoid arthritis patients (Lee et al., 2017). Halting IL-6 signaling *via* the monoclonal antibody sirukumab also reversed IL-6-mediated suppression of CYP3A, CYP2C9, and CYP2C19 activity in rheumatoid arthritis patients (Zhuang et al., 2015), suggesting that IL-6 is an important regulator of CYP enzymes.

#### 4.2.2 Tumor Necrosis Factor-α.

TNF-α enhances the induction of CYP1B1, whilst simultaneously suppressing benzo (a)pyrene-induced CYP1A1 expression in rat liver epithelial WB-F344 cells (Umannová et al., 2008). CYP3A11 and 3A25 were effectively down-regulated in mouse hepatocytes treated with TNF-α (Kinloch et al., 2011). In human hepatocytes, TNF-α down-regulated the gene expression of CYP1A1, CYP1A2, CYP2C8, CYP2D6, CYP2E1 and CYP3A4 (Abdel-Razzak et al., 1993; Aitken and Morgan, 2007; Dallas et al., 2012; Muntané-Relat et al., 1995). NF-κB was shown to play a significant role in CYP1A1 suppression caused by TNF-α and LPS (Ke et al., 2001).

A novel antagonist of soluble TNFα (XPro1595) selectively blocked the down-regulation of CYP3A11 and CYP3A25 mRNAs, as well as the induction of CYP2A4/5 in a C. *rodentium* model of infectious colitis (Nyagode et al., 2014). A recent study investigated the effects of TNF-α on CYP expression in hepatocytes from cynomolgus macaques, which showed significant reduction of CYP2C8 and CYP2C76 mRNA expression by TNF-α (Uno et al., 2020).

#### 4.2.3 Interleukin-1

Previous studies demonstrated that treatment of mice with IL-1, decreased CYPs contents (Bertini et al., 1989; Sujita et al., 1990). IL-1 rapidly suppressed CYP1A1 and CYP1A2 mRNA in rat hepatocytes and rabbit hepatocytes (Barker et al., 1992; Calleja et al., 1997). And CYP1A2, CYP2C8, CYP2E1, CYP3A, and CYP4A11 mRNA levels were down-regulated in human hepatocyte after IL-1β treatment (Abdel-Razzak et al., 1993; Dickmann et al., 2012). Immunoblot analysis of the CYP isozymes indicated that CYP2C11 and CYP3A were extensively reduced in IL-1β-induced fevered rat (Kihara et al., 1998). IL-1β significantly reduced CYP1A1, CYP2C8, CYP2C19, and CYP2C76 mRNA expression, but increased CYP3A5 mRNA expression in several cynomolgus hepatocytes (Uno et al., 2020). IL-1β also decreases phenobarbital- or bilirubin-mediated induction of CYP2B6, CYP2C9, CYP3A4 mRNA expression by negatively regulating CAR expression (Assenat et al., 2004). Lee et al. showed IL-1β down-regulated CYP3A expression at post-transcriptional level in a novel dual mode: nitric oxide (NO)- and proteasome-dependent at earlier time points and NO- and proteasome independent at later times (Lee et al., 2009).

#### 4.2.4 Interferon-γ

IFN-γ down-regulated the expression of CYP2D9, CYP2D22, CYP3A11, CYP3A25, and CYP4F18 mRNAs in a C. *rodentium* infection mice model and CYP2B9, CYP2D22, and CYP2E1 in a septic mice model (Nyagode et al., 2010). Furthermore, IFN-γ was shown to down-regulate CYP2E1 expression by suppressing native CYP2E1 promoter activity (Qiu et al., 2004). In human hepatocytes, the down-regulation of CYP1A2 and CYP3A4 expression by IFN-γ was observed (Donato et al., 1997). In male rat hepatocytes, IFN-γ reduced mRNA of CYP3A2 and CYP3A1, as well as CYP3A protein (Tapner et al., 1996).

### 4.3 Hepatic Injury Induced CYPs Alteration

Since the liver is one of the most affected organs in COVID-19 outside of the respiratory system, liver damage is common in COVID-19 patients (Fan et al., 2020). Previous study showed the expression or activity changed in hepatic injury. A reduction in CYP activity (CYP1A, 2C19 and 3A) was reported in cirrhosis (Villeneuve and Pichette, 2004). Acute experimental liver injury induced by CCl4, drastically reduced the activities of main liver CYP isoenzymes, such as CYP1A2, CYP2C6, CYP2E1 and CYP3A2 (Xie et al., 2014). Additionally, diminished expression and reduced enzymatic activity of CYP2E1, 3A11, 1A2, and 2C29 were found in drug-induced liver injury mice models (Bao et al., 2020). Consequently, COVID-19 associated haptic injury is likely to lead to changes in CYP expression and activity.

## 5 Altered CYPS in the Pathophysiology of COVID-19

**FIGURE 2:**
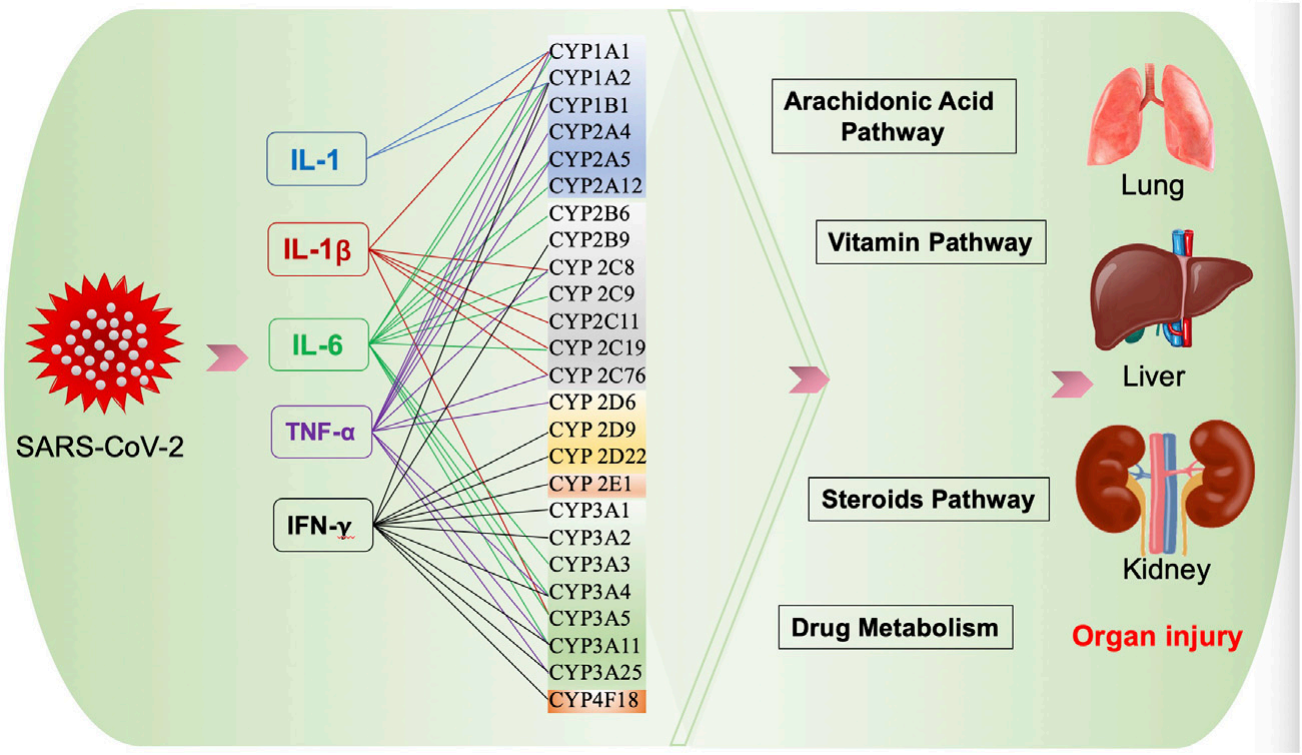
Possible effect of CYPs on COVID-19.

CYPs participate in the biosynthesis or catabolism of steroids, vitamins, eicosanoids, and fatty acids (Guengerich, 2008), which may be involved in the pathogenesis of COVID-19 (Figure 2).

### 5.1 Arachidonic Acid Pathway

AA is a polyunsaturated fatty acid produced from membrane phospholipids by phospholipase-A2 (PLA2) in inflammatory condition. AA-derived lipid autacoids, including prostaglandins (PGs), thromboxanes, and leukotrienes, are critical mediators in inflammation, and tissue homeostasis (Hoxha, 2020; Ripon et al., 2021). An integrated genomic-scale metabolic model of normal human bronchial epithelial cells (NHBE) infected with SARS-CoV-2, shows that AA metabolism was one of the most affected lipid metabolic pathways in SARS-CoV-2 infection (Nanda and Ghosh, 2021). One *in vitro* experiment revealed that AA metabolism was markedly perturbed by human coronavirus 229E (HCoV-229E) infection, and the exogenous supplement of AA in HCoV-229E-infected cells significantly suppressed HCoV-229E virus replication (Yan et al., 2019).

Aside from cyclooxygenase (COX) and lipoxygenase pathways, the CYP pathway is another important AA metabolism pathway (Ripon et al., 2021). CYP4A1 and CYP4A2 enzymes convert AA to hydroxyeicosatetraenoic acids (HETEs), which promote the expression of inflammatory cytokines and adhesion molecules (Ishizuka et al., 2008). Additionally, the CYP epoxygenase enzymes of CYP2C and CYP2J families generate epoxyeicosatrienoic acids (EETs) from AA, resulting in anti-inflammation, vasodilation, and pro-angiogenic effects (Iliff et al., 2010; Zhang et al., 2014). Multiple studies demonstrated that both EETs and HETEs play a role in lung injury and kidney injury (Hoff et al., 2019; Zhu et al., 2020). Consequently, it is considered that therapeutic strategies related to specific CYP inhibitors or inducers that improve AA metabolism may be beneficial in COVID-19 (Shoieb et al., 2020).

### 5.2 Vitamin Pathway

Vitamins are essential dietary components due to their antioxidant properties and immunomodulatory effects, which are beneficial in various infectious diseases, such as COVID-19 (Kumar et al., 2021a; Shakoor et al., 2021). A recent study evaluated the nutritional status of hospitalized COVID-19 patients aged 8–18 years, and results showed vitamin D deficiency in 82%, vitamin B12 deficiency in 18%, vitamin C deficiency in 17%, vitamin A deficiency in 13%, and vitamin E deficiency in 7% of patients (Karakaya Molla et al., 2021).

Vitamin A, also called retinoic acid (RA), exhibited a protective effect on HBV and measles virus infection (Li et al., 2018). Yuan et al. revealed that a retinoid derivative, is highly effective in interrupting the life cycle of Middle East respiratory syndrome (MERS) coronavirus and influenza A virus (Yuan et al., 2019).

Vitamin Bs are important for the normal physiological functioning of body cells (Kumar et al., 2021b). A recent study revealed the potential use of vitamin B9 (Folic acid) against SARS-CoV-2, after screening hundreds of nutraceuticals compounds against known SARS-CoV-2 therapeutic targets. Results indicate that vitamin B9 could contribute to fight against the COVID-19 pandemic (Kumar et al., 2021a).

Vitamin C is well known for its antiviral, antioxidant, anti-inflammatory and immunomodulating properties, which make it a potential therapeutic candidate against COVID-19 infection. Several recent studies show that most severe, or critically ill, COVID-19 patients had hypovitaminosis C, indicating that vitamin C can potentially be used as an adjunctive therapy in the critical care of COVID-19 patients (Arvinte et al., 2020; Chiscano-Camón et al., 2020; Holford et al., 2020).

Vitamin D helps to maintain calcium–phosphorus metabolism and inhibits the overexpression of inflammatory cytokines such as IL-1α, IL-1β, TNF-α (Hughes and Norton, 2009; Kumar et al., 2021b). Serum levels of vitamin D were also low in most of the critically ill COVID-19 patients admitted into intensive care units (ICU) (Arvinte et al., 2020).

COVID-19 may predispose to venous and arterial thrombosis disease due to excessive inflammation or hypoxia. Vitamin K1 could potentially help combat thrombotic complications in COVID-19 patients, due to its ability to activate the coagulation system. A clinical study has also shown that a low vitamin K status was associated with mortality in patients with COVID-19 (Linneberg et al., 2021).

Furthermore, multiple CYPs regulate vitamin metabolism. CYP26 enzymes are involved in the metabolism and elimination of vitamin A (Ross and Zolfaghari, 2011; Stevison et al., 2015). Research in both humans and a variety of animal species have revealed that several CYPs, such as CYP2R1, CYP27A1, CYP3A4, CYP2D25, CYP24A1, CYP27B1, and CYP11A1 are involved in vitamin D metabolism (Tuckey et al., 2008; Annalora et al., 2010; Wang et al., 2012; Jones et al., 2014; Wang et al., 2018a; Maksymchuk and Kashuba, 2020). CYP4F2 and CYP4F11 are both vitamin K1 and K2 ω-hydroxylases (Edson et al., 2013). In addition, CYP4F2 is the only human enzyme shown to metabolize vitamin E (Sontag and Parker, 2002; Bardowell et al., 2012). However, how CYP is involved in the pathophysiological process of COVID-19 through the vitamin pathway still needs further exploration.

### 5.3 Steroids

Recent advances suggest endocrine system dysfunction in COVID-19 patients. Angiotensin-converting enzyme 2 (ACE2) and transmembrane serine protease 2 (TMPRSS2) are expressed in several endocrine tissues, including the hypothalamus, pituitary, thyroid, adrenal, gonads, and pancreatic islets (Lazartigues et al., 2020; Puig-Domingo et al., 2020), suggesting that SARS-CoV-2 may invade and affect the endocrine system. SARS studies show that 39.3% survivors had evidence of hypocortisolism 3 months after recovery, and the majority of the hypothalamic–pituitary–adrenal (HPA) axis dysfunction resolved within a year (Leow et al., 2005). In the current COVID-19 pandemic, as well as in the SARS-CoV and MERS epidemics, females have a substantially lower mortality rate than males, which can be explained by sex differences in the response to inflammation and sex steroid hormones (Al-Lami et al., 2020; Leach et al., 2021).

On the other hand, CYPs are important for catalyzing specific reactions of steroid precursors in the steridogenic pathway (Ghayee and Auchus, 2007). CYP monooxygenase systems have been found to be involved in the process of arginine vasopressin (AVP)-induced adrenocorticotropic hormone (ACTH) secretion (Okajima and Hertting, 1986). Human CYP11B2 catalyzes the 11-hydroxylation of both progesterone and androstenedione (Glass et al., 2021), whilst CYP11B1 located in the zona fasciculata catalyzes the conversion of 11-deoxycortisol to cortisol (Portrat et al., 2001). CYP17A1 is required for the production of androgen and oestrogen precursors in the zona reticularis, testes and ovaries due to its 17a-hydroxylase activity and subsequent 17, 20-lyase activity (Ghayee and Auchus, 2007; Storbeck et al., 2015). CYP3A4 was the most efficient metabolic catalyst for several of the most frequently prescribed inhaled glucocorticoids (Moore et al., 2013). CYP3A5 activity in lung cells is also related to the metabolism of inhaled glucocorticoid fluticasone propionate, which increases the effective concentration at its target site (Murai et al., 2010). Additionally, CYP3A5 catalyzes 6β-hydroxylation of endogenous cortisol, which is associated with sodium and water retention in the kidney (Rais et al., 2013). Taken together, changes in CYPs may affect endocrine system function in COVID-19, but this still needs to be confirmed by a large number of future studies.

## 6 Possible Mechanism of CYPS Invovled in Organ Injury in COVID-19

Some COVID-19 patients, especially those with severe diseases, suffered from lung injury, kidney injury, even multi-organ failure. Numerous previous studies showed CYPs played a role in lung injury, kidney injury and liver injury, including CYPs we mentioned above that may affected in COVID-19, suggesting these CYPs may be involved in the pathophysiological process of severe COVID-19.

### 6.1 Acute Lung Injury

Lung is the main target of SARS-CoV-2, and lung injury is common in severe COVID-19 patients. Increasing studies showed CYPs play a role in ALI. A recent study found that CYP1A1 knockout enhanced LPS-induced ALI, as evidenced by increased IL-6, TNF-α, IL-1β in lung (Tian et al., 2021). CYP1A1 also protects mice models against hyperoxic lung injury by decreasing oxidative stress and susceptibilities to hyperoxia (Jiang et al., 2018; Lingappan et al., 2014; Lingappan et al., 2017), while CYP1B1 enzymes increase oxidative DNA adduct under hyperoxic conditions, contributing to lung injury. Additionally, CYP2E1 and CYP2A can also contribute to hyperoxic lung injury in ethanol and nicotine metabolism through oxidative stress pathway (Stading et al., 2021).

CYPs metabolizes AA to EETs and 20-hidroxyeicosatetranoic acids (20-HETEs), which is believed to play a protective role in lung injury (Stading et al., 2021). CYP4A and CYP4F, which are downregulated by inflammatory mediators (Nyagode et al., 2010; Dickmann et al., 2012), metabolize AA to 20-HETEs, which could also impact hyperoxic lung injury *via* the vasodilating effects of 20-HETEs (Stading et al., 2021).

### 6.2 Acute Kidney Injury

The incidence of AKI was about 8.9% in COVID-19 patients, but can reach 25% in critically ill patients (Chen et al., 2020; Gabarre et al., 2020). Current evidence suggests a link between CYPs and AKI. The expression and activity of CYP3A11 was predominant reduced in sepsis-AKI mice models (Sukkummee et al., 2019). CYP2C18, 2C19 expressions were significantly lower in uremic patients (Hu et al., 2018). And CYPs were shown to play an important role in metabolizing xenobiotics and thus reduce xenobiotics-induced renal toxicity (Xiao et al., 2008; Yao et al., 2014). Additionally, AA-derived CYP metabolites, EETs and 20-HETEs, play a key role in ischemia/reperfusion-induced AKI (Hoff et al., 2019; Zhu et al., 2020).

### 6.3 Hepatic Injury

In the current COVID-19 pandemic, hepatic dysfunction has been observed in 14–53% of patients, particularly in severe cases (Jothimani et al., 2020). Hepatic injury interacted with CYP expression and activity. Liver diseases cause changes in the expression and activity of CYPs, while CYPs also implicated in hepatic injury. Induction of CYP2E1 enzyme is known to play a role in the pathogenesis of alcoholic liver disease and thioacetamide induced-liver injury (Ramaiah et al., 2001; Stice et al., 2015). Several studies demonstrated that CYP2E1 inhibitor protects the liver against chemical-induced hepatic injury (Choi et al., 1996; Jeong, 1999; Lin et al., 2012).

## 7 Pharmacokinetics in COVID-19 Therapy

Since the primary function of CYP1-3 enzymes is facilitating drug metabolism, the main concern of the dysregulation of CYP expression in COVID-19 is the direct impact on drug disposition and pharmacokinetics in humans. Currently, there is no certified medication to treat COVID-19. Several drugs that are considered as potentially effective are being used in COVID-19 treatment, many of which are metabolized by CYPs. Of the total CYPs discovered to date, six of these are responsible for 90% of drug metabolism, including CYP1A2, CYP2C9, CYP2C19, CYP2D6, CYP2E1, and CYP3A4 (Gilani and Cassagnol, 2021). In this section, we aimed to summarize the role of these CYPs in COVID-19 drug therapy from the aspect of routine treatment, symptomatic support treatment and treatment of comorbidities (Table 2).

**TABLE 2:** CYPs involved in the metabolism of drugs in COVID-19 treatment.

CYP enzymes	Anti-viral drugs	Symptomatic and supportive treatment	Pharmacological therapy for comorbidity	Traditional Chinese medicine
CYP1A2	—	—	Clopidogrel clozapine, theophylline	Qingfei paidu decoction (1A family)
				Jingyin ranules (1A family)
CYP2B6	—	Propofol	Clopidogrel	—
		Diazepam		
		Tramadol		
CYP2C8	Remdesivir	Morphine	Pioglitazone	Qingfei paidu decoction
		Loperamide	Rosiglitazone	
		Ibuprofen	Repaglinide	Jingyin ranules
CYP2C9	—	Diazepam	Irbesartan	Qingfei paidu decoction
			Losartan	
			Nateglinide	
		Ibuprofen	Sulfonylureas	Jingyin ranules
			Clopidogrel	
		Celecoxib	Carvedilol	
			Warfarin	
CYP2C19	—	Diazepam	Indaparnide	Qingfei paidu decoction
		Omeprazole	Clopidogrel	Jingyin ranules
CYP2D6	Remdesivir	Tramadol	Propranolol	Qingfei paidu decoction
			Carvedilol	
	Chloroquine hydroxychloroquine	Loperamide	Diltiazem	
			Metoprolol	Jingyin ranules
			Nifedipine	
CYP2E1	—	Acetaminophen	Theophylline	Qingfei paidu decoction
				Jingyin ranules
CYP3A4	Lopinavir–ritonavir	Fentanyl	Indaparnide	Qingfei paidu decoction (3A family)
		Morphine		
		Midazolam	CCBs	
	Remdesivir	Alprazolam	Losartan	
		Tramadol		Jingyin ranules (3A family)
	Chloroquine hydroxychloroquine	Loperamide	Clopidogrel	
		Acetaminophen	Statin drugs	

Abbreviations: COVID-19, coronavirus 2019; CYP, cytochrome P450.

### 7.1 The Association Between Routine Drug Treatment and CYPs

#### 7.1.1 Lopinavir–Ritonavir

Upon referring to previous antivirus activity studies, lopinavir–ritonavir was proposed as an emergency treatment in COVID-19 (Magro et al., 2021). Lopinavir and ritonavir are both CYP3A4 substrates (Cvetkovic and Goa, 2003), so there is a potential for elevated levels following infection and inflammation-related down-regulation of CYP3A4 expression. This is supported by COVID-19 clinical pharmacokinetic data. Recent studies demonstrated that lopinavir trough concentrations were 3.5-fold higher in COVID-19 patients than in HIV-infected patients (Croxtall and Perry, 2010; Marzolini et al., 2020), which positively correlates with CRP values and was significantly lower when tocilizumab (IL-6 receptor antagonist) was pre-administered (Marzolini et al., 2020; Schoergenhofer et al., 2020).

#### 7.1.2 Remdesivir

Remdesivir is one of few Food and Drug Administration (FDA)-approved treatments for severe cases of COVID-19 (Tao et al., 2021). It is metabolized by both CYPs and non-CYP enzymes, and previous studies have demonstrated that remdesivir is a substrate for CYP2C8, CYP2D6, and CYP3A4 (Deb et al., 2021). Additionally, remdesivir also acts as an inhibitor of CYP1A2, CYP2C9, CYP2C19, CYP2D6, and CYP3A4 (Aleissa et al., 2020). Since CYP3A4 is a critical enzyme responsible for about 70% of the drugs that are clinically available (Deb and Arrighi, 2021), it should be noted that the suppression of CYP3A4 expression by concomitant inflammatory conditions and simultaneous application of other drugs metabolized by CYPs, could reduce the elimination of remdesivir and lead to unpredictable dose-toxicity.

#### 7.1.3 Chloroquine and Hydroxychloroquine

Chloroquine and hydroxychloroquine have been suggested as having an antiviral effect in COVID-19 patients, with side effects including arrythmias, cardiovascular complications, hepatological effects, and adverse vision effects (Cortegiani et al., 2020; Réa-Neto et al., 2021). Both of these drugs are metabolized by CYP3A4 and CYP2D6. Although CYP2D6 expression is not as affected by inflammatory factors as CYP3A4, the highly frequent polymorphic presence of CYP2D6 could also lead to modified elimination of these drugs and eventually life-threatening drug-adverse effects (Nefic, 2018; Deb et al., 2021). However, hydroxychloroquine plasma concentrations appear to have no correlation with CRP values in COVID-19 patients (Marzolini et al., 2020).

#### 7.1.4 Corticosteroids

Corticosteroids are widely used in the treatment of COVID-19 due to their anti-inflammatory and immunosuppressive effects (Wang et al., 2021). Corticosteroids are substrates and inducers for CYP3A4 (Gentile et al., 1996; Mccune et al., 2000). Glucocorticoids are predominantly metabolized by CYP3A, and their plasma concentrations are influenced by CYP3A activity (Varis et al., 1998; Varis et al., 2000a; Varis et al., 2000b). Low hepatic CYP3A activity caused by hyperinflammation in COVID-19 may significantly contribute to the risk of glucocorticoid-related complications, such as steroid-induced osteonecrosis of the femoral head (Kaneshiro et al., 2006). However, glucocorticoids at doses used clinically also increased CYP3A4 activity, but with extensive intersubject variability (Mccune et al., 2000). Therefore, due to the heterogeneity of the induction effect of glucocorticoids on CYP activity, whether it can counteract the suppression effect of CYP expression and activity caused by inflammatory mediators may vary among individuals.

#### 7.1.5 Symptomatic and Supportive Treatment

The symptoms of COVID-19 patients are varied, with the most common being fever, cough, digestive tract symptoms, sleep disorders and headaches, which are often treated by medication (Guan et al., 2020a; Bhat and Chokroverty, 2021; Fernández-De-Las-Peñas et al., 2021). Ibuprofen and acetaminophen are the most commonly used antipyretics. Ibuprofen metabolism is strongly linked to CYP2C8 and CYP2C9 (García-Martín et al., 2004), whilst CYPs (CYP3A4, CYP2E1) have some role mainly at toxic concentrations of acetaminophen. CYP3A4 is the major CYP enzyme involved in acetaminophen bioactivation. Alprazolam is a CYP3A4 substrate, often prescribed to treat COVID-19 patients with sleep disorders (Boulenc et al., 2016; Sánchez Díaz et al., 2021). Proton pump inhibitors (PPI), commonly used drugs for gastrointestinal diseases, are metabolized by CYP2C19 (Zvyaga et al., 2012). Celecoxib, a cyclooxygenase (COX)-2 inhibitor, is the substrate of CYP2C9 (Chan et al., 2009), and tramadol is a substrate of CYP2D6 (Xu et al., 2014), and therefore both can be used to treat headaches (Piovesan et al., 2002; Robbins, 2004).

ARDS is a major complication in severe COVID-19 patients, when analgesics and sedatives are routinely used for patients on mechanical ventilation. Fentanyl and morphine are also commonly used for analgesia in patients on mechanical ventilation. Fentanyl and sufentanil are metabolized by CYP3A4 (Tateishi et al., 1996; Labroo et al., 1997), and hepatic CYP3A4 and CYP2C8 are the main CYPs responsible for morphine N-demethylation (Projean et al., 2003). Propofol, benzodiazepine and dexmedetomidine are commonly used for sedation. Propofol is metabolized by CYP2B6 (Murayama et al., 2007; Oshio et al., 2019), whilst the most commonly used benzodiazepine drug Midazolam is metabolized by CYP3A4 and serves as a probe for CYP3A catalytic activity (Olkkola and Ahonen, 2008; Nassi et al., 2020). It has been reported that CYPs (mainly CYP2A6) primarily mediated aliphatic hydroxylation of dexmedetomidine, generating 3-hydroxy dexmedetomidine and other metabolites, in human liver microsomes (Weerink et al., 2017; Wang et al., 2018b). Most of these medications can be extremely harmful if plasma levels are increased following a lack of metabolism by inflammation-mediated CYP3A4 or CYP2B6 suppression.

### 7.2 Pharmacological Therapy for Comorbidity

Elderly patients have the highest mortality rate amongst COVID-19 patients, which is generally related to co-existing underlying diseases. Similar to most studies, our previous data showed that the most common comorbidities in this population were hypertension, coronary heart disease, and diabetes (Guan et al., 2020b; Huang et al., 2020; Wang et al., 2020). Many drugs used to treat chronic diseases are also metabolized by CYPs.

Anti-hypertensive drugs incorporate several classes: diuretics, angiotensin-converting enzyme (ACE) inhibitors, calcium channel blockers (CCBs), angiotensin II receptor blockers (ARBs), and beta-blockers (Peyriere et al., 2012). Indaparnide, a long-acting thiazide-related diuretic, is metabolized by CYP3A4 and CYP2C19 (Yan et al., 2012). Several beta-blockers, such as propranolol, are largely metabolized by CYP2D6 (Peyriere et al., 2012). All CCBs are substrates for CYP3A4. Losartan, the leading ARB, is bioactivated by CYP2C9 and subsequently metabolized by CYP3A4. Another ARB, irbesartan, is metabolized by CYP2C9 (Peyriere et al., 2012).

Anti-platelet and anti-coagulant drugs are commonly used in the treatment of cardiovascular diseases. CYP2C19, CYP1A2, and CYP2B6 catalyze clopidogrel to the immediate precursor of its pharmacologically active metabolite, whilst CYP3A4, CYP2B6, CYP2C19, and CYP2C9 contribute to the active metabolite formation (Kazui et al., 2010). CYP2C9 is responsible for warfarin metabolism (Mikheeva et al., 2008).

Several oral antidiabetic drugs are also metabolized by CYPs. For example, pioglitazone and rosiglitazone are metabolized mainly by CYP2C8, whilst sulfonylureas are mainly metabolized by CYP2C9; and to a lesser extent by CYP3A4 (Holstein and Bell, 2009). Repaglinide is metabolized mainly through CYP2C8 whereas nateglinide metabolism predominantly involves CYP2C9 (Holstein and Bell, 2009).

A recent study of 227 hospitalized COVID-19 patients showed that 38% had at least one clinically significant potential drug–drug interaction. More than half of the interactions were between lopinavir/ritonavir and regularly prescribed medications for the management of comorbidities or COVID-19 symptoms (Mahboobipour and Baniasadi, 2020). As CYPs are the most important family of drug metabolism enzymes, the interactions between drugs metabolized by CYPs need to be carefully considered by clinicians.

Additionally, traditional Chinese medicine is widely used in the treatment of COVID-19 in China (Liang et al., 2021; Wu and Zhong, 2021). Jingyin granules and Qingfei Paidu decoction, have been recommended for treating the H1N1 influenza A virus infection and COVID-19 in China, and have exhibited an inhibitory effect on CYP1A, CYP2A6, CYP2C8, CYP2C9, CYP2D6, CYP2E1, CYP2C19 and CYP3A (Zhang et al., 2021b; Zhang et al., 2021c).

## 8 Conclusion

The recent emergence of the COVID-19 pandemic has caused unprecedented global healthcare problems. The most striking pathophysiological feature of COVID-19 is the state of excessive inflammatory response. As the most common drug metabolizing enzyme family, CYPs are closely related to the metabolism of endogenous and exogenous substances. In this review, we analyzed and summarized current evidences regarding the possible changes and roles of CYPs in COVID-19. In COVID-19, viral infection, excessive inflammatory response, and hepatic impairment may all affect CYP expression. CYPs may influence the pathophysiological process of COVID-19 through AA, vitamins, and steroid pathways. Moreover, many of the drugs that are likely to be used in COVID-19 patients are metabolized by CYPs. Since the expression of CYPs may be greatly altered in COVID-19 patients, drug pharmacokinetics may also vary, and drug-related side effects may increase in these patients. In the case of co-administration of multiple drugs, the risk of drug interactions may even increase, and therefore, monitoring of drug concentrations and side effects is essential in this population. Overall, the information on the relationship between COVID-19 pathophysiology and CYPs status, will potentially minimize drug-related toxicity and optimize the treatment of infected individuals.

## Glossary

AA: Arachidonic acid
ACE: Angiotensin-converting enzyme
ACTH: Adrenocorticotropic hormone
AhR: Aryl hydrocarbon receptor
AKI: Acute kidney injury
ARBs: Angiotensin II receptor blockers.
ARDS: Acute respiratory distress syndrome
AVP: Arginine vasopressin
CAR: Constitutive androstane receptor
CCBs: Calcium channel blockers
COVID-19: Coronavirus disease 2019
COX: Cyclooxygenase
CYP: Cytochrome P450
EETs: Epoxyeicosatrienoic acids
HCV: Hepatitis C virus
HETEs: Hydroxyeicosatetraenoic acids
HPA: Hypothalamic–pituitary–adrenal axis
ICU: Intensive care units
IFN: Interferon
IL: Interleukin
LPS: Lipopolysaccharide
MERS: Middle East respiratory syndrome
NHBE: Human bronchial epithelial cells
PGs: Prostaglandins
PLA2: Phospholipase-A2
PXR: Pregnane X receptor
RA: Retinoic acid
SARS-CoV-2: Severe acute respiratory syndrome coronavirus 2
TMPRSS2: Transmembrane serine protease 2
TNF: Tumor necrosis factor
WHO: World Health Organization
